# Theoretical impact of insecticide-impregnated school uniforms on dengue incidence in Thai children

**DOI:** 10.3402/gha.v6i0.20473

**Published:** 2013-03-28

**Authors:** Eduardo Massad, Marcos Amaku, Francisco Antonio Bezerra Coutinho, Pattamaporn Kittayapong, Annelies Wilder-Smith

**Affiliations:** 1 School of Medicine, University of São Paulo, São Paulo, Brazil; 2 London School of Hygiene and Tropical Medicine, London, UK; 3 School of Veterinary Medicine, University of São Paulo, São Paulo, Brazil; 4 Center of Excellence for Vectors and Vector-Borne Diseases, Faculty of Science, Mahidol University at Salaya, Nakhon Phatom, Thailand; 5 Department of Public Health and Clinical Medicine, Centre for Global Health Research, Umeå University, Umeå, Sweden; 6 Department of Medicine, National University of Singapore, Singapore; 7 Emerging Infectious Diseases Program, Duke-NUS Graduate Medical School, Singapore

**Keywords:** dengue, school children, impregnated clothes, insecticide-treated uniforms, force of infection, mathematical models, Thailand

## Abstract

**Background:**

Children carry the main burden of morbidity and mortality caused by dengue. Children spend a considerable amount of their day at school; hence strategies that reduce human–mosquito contact to protect against the day-biting habits of *Aedes* mosquitoes at schools, such as insecticide-impregnated uniforms, could be an effective prevention strategy.

**Methodology:**

We used mathematical models to calculate the risk of dengue infection based on force of infection taking into account the estimated proportion of mosquito bites that occur in school and the proportion of school time that children wear the impregnated uniforms.

**Principal findings:**

The use of insecticide-impregnated uniforms has efficacy varying from around 6% in the most pessimistic estimations, to 55% in the most optimistic scenarios simulated.

**Conclusions:**

Reducing contact between mosquito bites and human hosts via insecticide-treated uniforms during school time is theoretically effective in reducing dengue incidence and may be a valuable additional tool for dengue control in school-aged children. The efficacy of this strategy, however, is dependent on the compliance of the target population in terms of proper and consistent wearing of uniforms and, perhaps more importantly, the proportion of bites inflicted by the *Aedes* population during school time.

Dengue is the most important mosquito-borne disease of humans that has not only increased in numbers but also expanded geographically (1–3). Global reports of dengue haemorrhagic fever (DHF) have increased five-fold in the past 20 years (4). Some 3.6 billion people, half of the world's population, are now at risk (1). The World Health Organization (WHO) estimates that there are 50–100 million infections worldwide every year, of which 21,000 are fatal (3). The disease is now endemic in more than 100 countries in Asia, Africa, and the Americas with south-east Asia being the most seriously affected. Children carry the main burden of morbidity and mortality (5). Dengue infections in children cause large disruptions in schooling, and loss in parental wage earning (6). According to the WHO, the number of disability-adjusted life years (DALYs) worldwide is estimated to range between 528 and 621 per million population (7). Preliminary adjustment for under-reporting could raise this total to $1.8 billion, and incorporating costs of dengue surveillance and vector control would raise the amount further (8, 9).

Dengue viruses are transmitted by mosquitoes of the genus *Aedes*, subgenus *Stegomyia*. The principal vector, *Aedes aegypti (Stegomyiaaegypti)*, is now well established in much of the tropical and subtropical world, and as a peri-domestic species, it is particularly well established in urban and semi-urban areas, thriving on proximity with humans. It typically breeds in clean stagnant water, particularly in artificial containers, and is therefore well adapted to urban life. *Aedes* is a daytime feeder (3), which means that strategies need to be employed to prevent mosquito–human contact during the daytime.

Current control strategies are clearly insufficient to combat dengue in endemic countries. Vector control (both adulticidal and larvicidal) has shown some impact but for various reasons has not resulted in the significant reduction of dengue (10, 11). There is an urgent need to evaluate additional control strategies, in particular, for children.

We previously hypothesized that impregnated school uniforms could be a potential strategy to reduce dengue infections in school-aged children (12). Because children of this age group spend a considerable amount of their day at school, the rationale is to develop strategies that reduce human–mosquito contact to protect against the day-biting habits of *Aedes* mosquitoes at schools. School uniforms are a cultural norm in many developing countries and are worn throughout the day on an almost daily basis. Insecticide-treated school uniforms may therefore result in a reduction of dengue infections in school-aged children.

To test this hypothesis, school-based intervention trials at various settings are necessary. Such a large community-based trial is currently in preparation in Thailand (13, 14). However, such trials are costly and difficult to implement and it will take some years before the results will be available. We set out to estimate the potential impact of impregnated school uniforms on the incidence of dengue in school-aged children using mathematical modelling.

## Methods

### SI models

We first deduced an approximate risk for dengue acquisition in a stochastic framework.

Assuming a closed population of size *N* in the absence of competitive risks, the risk of dengue can be stochastically calculated by a two-state model without recovery, that is, a SI type model, in which ‘*S*’ individuals are susceptible to dengue and ‘*I*’ individuals are those who have acquired the infection once in the past, remaining protected for life.

We calculated the probability that *y* individuals are in the state *I* in the period of time between *t* and *t*+Δ*t* using the following equation (in preparation):

1 $$P_{y}^{I}(t+\Delta t)=P_{y}^{I}(t)(1-\lambda x\Delta t)+P_{y-1}^{I}\lambda \left(x+1\right)\Delta t$$

In eq. (1), the first term refers to the probability that there were *y* individuals at time *t* in the *I* condition and that no susceptible individuals *x* acquired the infection in the period. The second term refers to the probability that there were (*y* – 1) individuals at time *t* in the *I* condition and that one susceptible individual *x* acquired the infection in the period.

From eq. (1), it is possible to obtain:

2 $$\frac{{dP}_{y}^{I}(t)}{dt}=-\lambda \left(N-y\right)P_{y}^{I}(t)+\lambda \left(N-y+1\right)P_{y-1}^{I}$$

Note that in eq. (2) we are assuming the closed population *N*=*x*+*y*.

The general expression for the probability generation function (PGF), G(*z,t*), is given by:

3 $$G(z,t)=\sum_{y=0}^{N}z^{y}P_{y}^{I}(t)$$

For the particular model expressed in eq. (2), it is possible to calculate the PGF such that:

4 $$G(z,t)={\left[z-(z-1)\exp(-\lambda t)\right]}^{N}$$

Now the average number of *y* individuals at time *t* can be calculated by taking the first partial derivative of the PGF with respect to *z* at *z*=1:

5 $${\frac{\partial G(z,t)}{\partial z}|}_{z=1}=N\left[1-\exp(-\lambda t)\right]$$

Hence, the average probability π_*d*_(*t*) of at least one infection with the dengue virus at time *t* is given by:

6 $$\pi_{d}(t)=\left[1-\exp(-\lambda t)\right]$$

The variance of the probability distribution for the number of infected individuals at time *t* is given by:

7 $${N\sigma^{2}=\frac{\partial^{2}G(z,t)}{\partial z^{2}}|}_{z=1}+{\frac{\partial G(z,t)}{\partial z}|}_{z=1}-{\left[{\frac{\partial G(z,t)}{\partial z}|}_{z=1}\right]}^{2}$$

which results in:

8 $$\sigma^{2}(t)=\exp(-\lambda t)\left[1-\exp(-\lambda t)\right]$$

Therefore, the probability of dengue acquisition at time *t* is given by:

9 $$\pi_{d}(t)=\left[1-\exp(-\lambda t)\right]\pm 1.96\left\{\sqrt{\frac{\exp(-\lambda t)\left[1-\exp(-\lambda t)\right]}{N}}\right\}$$

where the second term is the 95% confidence interval.

### Force of infection

The force of infection, λ, is the per capita incidence rate and is defined as (15–17):

10 $$\lambda =ab\frac{I_{M}}{N_{H}}$$

where, a is the mosquitoes’ biting rate, b is vector competence (the probability that a bite from an infected mosquito results in a new case), *I*_M_ is the number of infected mosquitoes and *N*_H_ is the total number of humans hosts.

Actually, dengue infection can be caused by any of the four known serotypes. Cross-immunity (if any) is short-lived such that each dengue infection can be considered independently, although the phenomenon of antibody enhancement is known to be involved in haemorrhagic dengue. Therefore, the total force of infection *λ*_*T*_ can be expressed as:

11 $$\lambda_{T}=\frac{a}{N_{H}}\sum_{i=1}^{4}b_{i}I_{{\text{M}}_{i}}$$

where *b*_*i*_ and *I*_M_*i*__ are the serotype-specific transmissibility and number of infected mosquitoes, respectively (note that the biting rate is considered to be equal for all serotypes). Hence, the probability of infection by any serotype is now

12 $$\pi_{d}(t)=\left[1-\exp(-\lambda_{T}t)\right]$$

Since our hypothesis is that the use of impregnated school uniforms reduces the contact between the hosts and the mosquitoes’ bites, it suffices to estimate the sensitivity of the force of infection, λ to the mosquitoes’ biting rate, a, which, as mentioned above, is assumed to be the same for all the four serotypes.

To estimate the sensitivity of a model variable in steady state, *V*_*i*_, to a parameter *θ*_*j*_, we consider the relative variation in the parameter, $\frac{\Delta \theta_{j}}{\theta_{j}}$. This variation will correspond to a variation $\frac{\Delta V_{i}}{V_{i}}$ in the model variable *V*_*i*_ given by (18):

13 $$\frac{\Delta V_{i}}{V_{i}}=\frac{\theta_{j}}{V_{i}}\frac{\partial V_{i}}{\partial \theta_{j}}\frac{\Delta \theta_{j}}{\theta_{j}}$$

Hence, the sensitivity of λ to the biting rate a is given by:

14 $$\frac{\Delta \lambda }{\lambda }=\frac{a}{\lambda }\frac{\partial \lambda }{\partial a}\frac{\Delta a}{a}$$

and

15 $$\frac{\partial \lambda }{\partial a}=b\frac{I_{M}}{N_{H}}+ab\frac{\partial }{\partial a}\left[\frac{I_{M}}{N_{H}}\right]$$

The second term of eq. (15) refers to the influence of biting rate on the infected mosquito density as related to human hosts. Since the proposed strategy is aimed only at a small sample of school children, this term can be neglected, such that eq. (15) is reduced to $\frac{\partial \lambda }{\partial a}=b\frac{I_{M}}{N_{H}}$. Now, assuming a typical biting rate for *Aedes aegypti* as *a*=0.164 days^−1^ (19); an average probability of infection from mosquitoes to humans as *b*=0.6 (20); an average infected mosquitoes’ density as $\frac{I_{M}}{N_{H}}=0.00143$[calculated from the estimations for Thailand by Massad, Roklov and Wilder-Smith (21)]; and an estimated total force of infection (see below) as λ=0.000141 cases per person-years, we end up with a sensitivity of one percentage point reduction in λ for each percentage point reduction in *a*.

In order to test the hypothesis that the use of insecticide-impregnated school uniforms will reduce the dengue's force of infection, we consider the probability of dengue acquisition as equal to the proportion of seropositive hosts at time *t*, $s_{H}^{+}(t)$. Therefore, in the absence of competitive risks this has the approximate dynamics as in eq. (9):

16 $$s_{H}^{+}(t)=1-\exp(-\lambda t)\pm 1.96\sqrt{\frac{\exp(-\lambda t)\left[1-\exp(-\lambda t)\right]}{N}}$$

The efficacy of the strategy at time *t* can be calculated according to (22):

17 $$\mathrm{Eff}(t)=1-\frac{{\pi_{d}(t)|}_{1}}{{\pi_{d}(t)|}_{0}}$$

where the subscripts 0 and 1 denote the probabilities of infection before and after the introduction of the control strategy, respectively.

## Results

Calculating the partial derivative of λ with respect to the biting rate a with eq. (4) has the following results: for each 1% variation in the biting rate a results in a variation of about 5% in the force of infection λ. Therefore, reducing the contact between mosquitoes’ bites and the human hosts is theoretically very effective in reducing the infection's incidence.

Using eq. (14), if we assume the annual incidence of dengue infection is 5% in Thai children (14, 23), the force of infection at this moment is 1.41×10^−4^ cases per person-years. Assuming that a given proportion of children are expected to wear the insecticide-impregnated clothes during their school period, and the proportion of school time (that is the school time in hours divided by 24 hours) children wear the uniform, we can estimate the impact of this strategy as dependent on the proportion of all the mosquitoes’ bites that occur during that period. Then, the expected proportion of seropositive some year after the intervention is calculated as:

18 $$s_{H}^{+}\left(365days\right)=1-e^{\left[1-\left(1.41\times 10^{-4}(1-(A*B))\right)365\right]}\pm 1.96\sqrt{\frac{e^{\left[-\left(1.41\times 10^{-4}(1-(A*B))\right)365\right]}\left[1-e^{\left[-\left(1.41\times 10^{-4}(1-(A*B))\right)365\right]}\right]}{2,012}}$$

where 2,012 is the sample size of school children in the randomized controlled trial that is currently being conducted in eastern Thailand (14), *A* refers to a combination of the proportion of children that wear the uniform, the probability that the mosquitoes will get into contact with the insecticide-impregnated uniform and the proportion of school time children wear the uniform, while *B* is the proportion of mosquito bites that occur during school time.

**Fig. 1:**
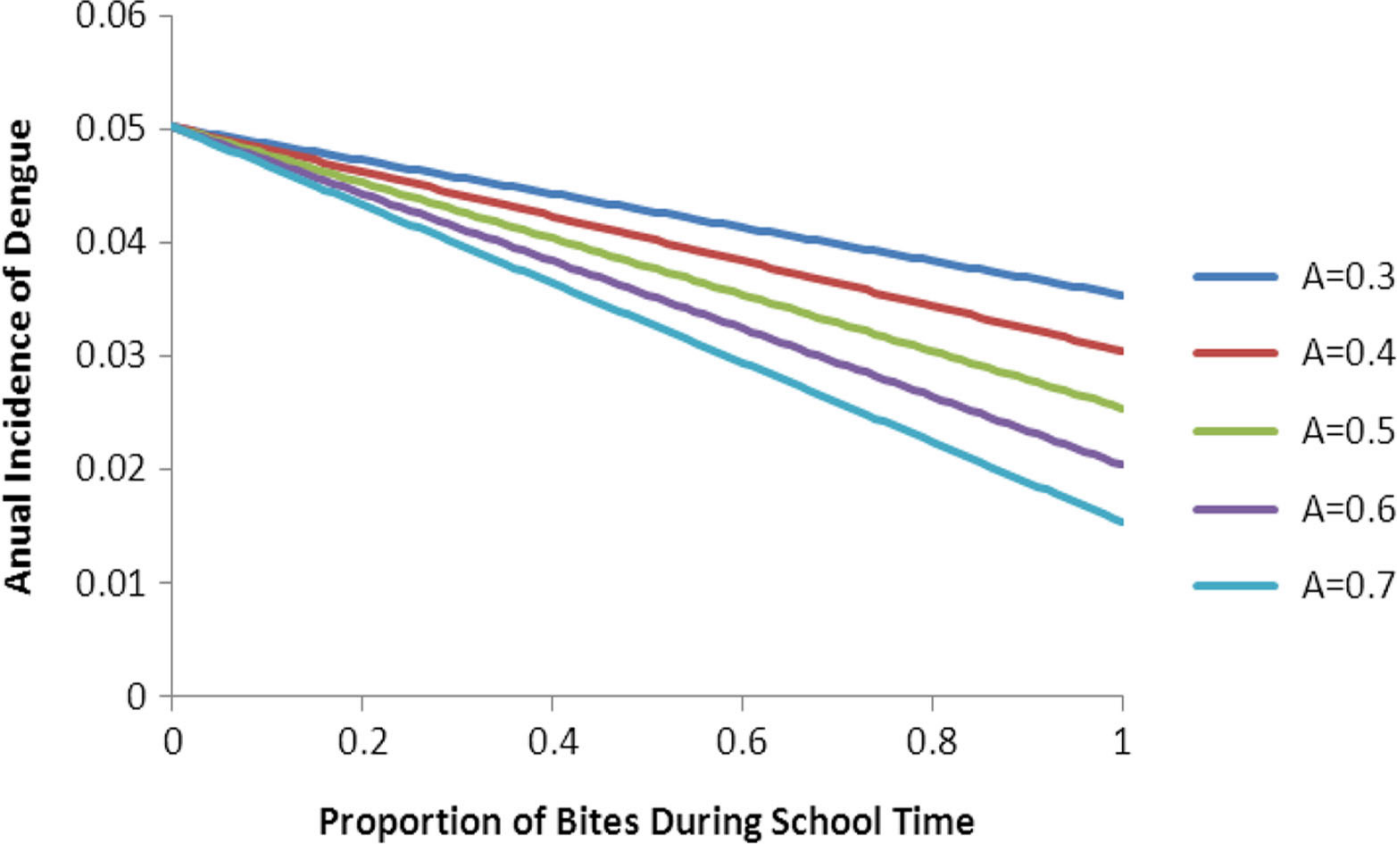
Projected scenarios from the numerical simulations of eq. (18). The figure shows only the mean annual incidence as a function of the proportion of bites during school time [parameter *B* in eq. (18)].

Fig. 1 shows the impact of the strategy on the proportion of seropositive children after 1 year of the intervention as a function of *B*.

In Fig. 1, we consider various realities that combine the facts that efficacy is never 100%, as the knock down effect is around 80% in ideal laboratory conditions, but will theoretically be much lower in real world conditions where uniforms do not cover the full body, uniforms are not worn the whole day, and uniforms are not worn during the weekends. We therefore simulated five scenarios varying parameter in eq. (18) from 0.3 to 0.7.

Another way to see the impact of the proposed strategy is shown in Table 1. In the table we show the result of the simulation of eq. (18) for some selected values of parameters B and A. Efficacy was calculated according to eq. (17).

**Table 1:** Efficacy of impregnated uniforms for selected values of parameters *B* and *A*

*B*	*A*=0.3	*A*=0.4	*A*=0.5	*A*=0.6	*A*=0.7
20%	5.9%	7.8%	9.8%	11.7%	13.7%
40%	11.7%	15.7%	19.6%	23.5%	27.5%
60%	17.6%	23.5%	29.5%	35.4%	41.4%
80%	23.5%	31.4%	39.4%	47.4%	55.4%

Calculated according to eq. (17).

Note that the use of insecticide-impregnated uniforms has efficacy varying from around 6% in the most pessimistic, up to 55% in the most optimistic scenarios simulated.

## Discussion

The mass distribution of insecticide-treated bed nets as part of the Roll Back Malaria (RBM) partnership is expected to contribute majorly towards successful malaria control (24). The current target of universal access to long-lasting, insecticide-treated nets is 80% coverage which aims to reduce malaria deaths by 75% by 2015 (25).The cost-effectiveness and general applicability of insecticide-treated bed nets to control malaria has turned this strategy into a sort of gold-standard of malaria control (26). The protective efficacy of insecticide-treated bed nets, however, varies according to the coverage achieved: protective efficacy was demonstrated to be 68% in communities with an average insecticide-treated bed nets coverage of 50%; 31% in communities with an insecticide-treated bed nets coverage of 16–30%; and no protective efficacy in communities with insecticide-treated bed nets coverage below 16% (27).

In another study, Vanlerberghe et al. (28) deployed insecticide-treated window curtains in households and showed that this strategy can result in significant reductions in *Aedes aegypti* levels when dengue vector infestations are moderate.

Hence, the use of insecticide-impregnated fabric as a repeller/barrier between mosquitoes and human hosts is already established as an effective tool against vector-borne infections. Using mathematical models, it is possible to define rational expectations about insecticide-impregnated clothes to control dengue, in the same way these models were used to quantify the impact of the use of bed nets to control malaria (24).

Our findings are consistent with those described by Amaku et al. (2012, Biosystems, available at: http://arxiv.org/abs/1302.1166) where they presented a sensitivity analysis of the impact of four vector-control strategies on the basic reproduction number, *R*_*0*_, and on the force of infection, λ, of dengue. They conclude that of the available vector-control strategies adulticide application is the most effective, followed by reduction of contact with the mosquito's bites, search and destroying breeding places, and, finally, larvicides.

Here we have shown that reducing the contact between mosquitoes’ bites and the human hosts via insecticide-treated uniforms during the daytime is theoretically very effective in reducing the infection's incidence. Of course the efficacy of this strategy is very much dependent on the compliance of the target population in properly and consistently wearing the uniforms, and, perhaps more importantly, the proportion of bites the *Aedes* population inflicts during school time [parameters *A* and *B* in eq. (15)]. The value of such a strategy also depends on whether such impregnated uniforms are culturally acceptable and that no skin irritations or other adverse events will occur. We will therefore need to wait for the results of a school-based randomized control trial that is currently being conducted in Thailand to know the effectiveness of such a strategy under field conditions (14). Also of concern is the possibility of evolution of resistance against the insecticide, a phenomenon already observed in the impregnated bed nets used against the malaria mosquitoes (29, 30). This highlights the need to sustain research on discovering new insecticides (31) And to test insecticide combinations in the same fashion as is applied to avoid the evolution of antibiotic and/or antiviral resistance. Waning of efficacy over time after repeated washing is another issue that would need to be studied under field conditions.

One important point to comment is that at first sight it may seem that the sensitivity of the force of infection is dependent on the ratio of infected mosquitoes to the total human population, $\frac{I_{\text{M}}}{N_{H}}$. This ratio, however, is almost linearly related to the human prevalence of dengue $\frac{I_{\text{M}}}{N_{H}}$(Amaku et al., 2012, Biosystems, submitted), which in turn is almost linearly related to the force of infection. Therefore, when the ratio $\frac{I_{\text{M}}}{N_{H}}$increases, the human prevalence and the force of infection increase by almost the same amount. Hence, the term $\frac{a}{\lambda }$in eq. (12) decreases by practically the same amount as the term $\frac{\partial \lambda }{\partial a}$increases and the effect is cancelled out.

Finally, an apparent limitation of the model is the linearity in eqs. (1) and (2) with respect to the force of infection λ. This is justified by the assumption that the small cohort of children in the experiment is subjected to the force of infection of their original population but that this number is too small to influence it.

In summary, based on mathematical modeling, the use of insecticide-impregnated uniforms has an efficacy varying from around 6% in the most pessimistic scenarios, to 55% in the most optimistic scenarios simulated. If such a strategy is shown to be safe and culturally acceptable in field conditions, then even a theoretical reduction of dengue incidence by only 6 to 55% may justify implementing this approach to control dengue in school aged children.
